# Long-Term Fenofibrate Treatment Stimulates the Phenotypic Microevolution of Prostate Cancer Cells In Vitro

**DOI:** 10.3390/ph15111320

**Published:** 2022-10-26

**Authors:** Karolina W. Warzecha, Maciej Pudełek, Jessica Catapano, Zbigniew Madeja, Jarosław Czyż

**Affiliations:** Department of Cell Biology, Faculty of Biochemistry, Biophysics and Biotechnology, Jagiellonian University, ul. Gronostajowa 7, 30-387 Cracow, Poland

**Keywords:** fenofibrate, prostate cancer, microevolution, drug resistance

## Abstract

Fenofibrate is a widely used anti-hyperlipidemic agonist of peroxisome proliferator-activated receptor alpha (PPARα). As a metabolic blocker, fenofibrate interferes with cancer promotion/progression via its misbalancing effects on cellular metabolism. However, the consequences of its long-term application for patients with diagnosed drug-resistant cancers are unknown. We addressed this point by tracing the phenotypic microevolution of naïve and drug-resistant prostate cancer PC3_DCX20 cells that underwent a long-term exposition to 10 μM and 50 μM fenofibrate. Their resistance to fenofibrate, metabolic profile and invasive phenotype were estimated in the control conditions and under fenofibrate-induced stress. Apparently, drug efflux systems are not effective against the cytostatic FF action. However, wtPC3 and PC3_DCX20 cells that survived the long-term 50 μM fenofibrate treatment gave rise to lineages that displayed an increased proliferation rate, lower motility in the control conditions and enhanced fenofibrate resistance. Attenuated fenofibrate bioavailability modified the pattern of PC3 microevolution, as illustrated by phenotypic differences between wtPC3/PC3_DCX20 lineages propagated in the presence of 50 μM and 10 μM fenofibrate. Collectively, our observations indicate that fenofibrate acts as a selective factor that affects prostate cancer microevolution. We also pinpoint potential consequences of long-term exposition of prostate cancer patients to metabolic blockers.

## 1. Introduction

Cancer drug resistance is the most prominent current problem in oncology. In response to chemotherapeutic stress, originally drug-sensitive cancer cells can activate multiple mechanisms to remove or inactivate the chemotherapeutics and/or to reduce the consequences of their action. These systems comprise the drug efflux pumps that remove the xenobiotics from the cytoplasm [1,2] and the drug metabolism pathways that inactivate the drugs and attenuate their activity [3]. Additionally, repair systems (i.e., chaperoning/autophagy) are activated that counteract the adverse effects of xenobiotics [4,5]. Consequently, long-term chemotherapeutic stress results in the selective expansion/microevolution of the cell lineages that are characterised by a stably enhanced drug resistance [6]. Since this process is often correlated with multipotency (including the multipotency of cancer stem cells; CSCs [2,6]) or an increased invasiveness of cancer cells (due to the epithelial–mesenchmal transition, EMT [7]), tumors generally reoccur in a more aggressive form after the end of the chemotherapeutic procedure. On the other hand, drug resistance systems usually require energy to act effectively. Accordingly, anti-cancer strategies have been proposed based on the combined application of cytostatic(s) and metabolic blockers that interfere with the function of drug resistance systems [8,9]. Amongst the metabolic blockers that are potentially suitable for combined therapies for drug-resistant tumors, fenofibrate has recently gained attention as a systemically neutral and effective anti-cancer agent [10].

Fenofibrate (FF) is an FDA-approved drug that is commonly applied in anti-hyperlipidemic therapies for elderly patients [11]. As a peroxisome proliferator-activated receptor alpha (PPARα) agonist, FF increases lipolysis in an array of tissues. In the liver, FF controls lipid and carbohydrate metabolism via lipoprotein lipase activity, significantly decreasing triglyceride levels in the blood. Concomitantly, it improves lipoprotein remnant clearance, significantly lowers low-density lipoprotein (LDL) synthesis and raises high-density lipoprotein (HDL) levels [12,13]. Along with a nitric oxide (NO)/prostaglandin-mediated endothelial normalization, FF provides cardiovascular protection [14,15,16]. It also exhibits potential therapeutic efficacy for nonalcoholic fatty liver disease [13,17] and may interfere with the development of early diabetic testicular symptoms and retinopathy in a PPARα-independent manner [18,19].

Apart from this canonical activity, the cytostatic/cytotoxic effects of FF have been observed in an array of cancer models [10,20,21,22,23]. They apparently result from the misbalancing effects of FF on the metabolism of cancer cells. FF has been shown to interfere with the mitochondrial machinery of cancer cells, where it apparently prompts intracellular oxidative stress [20]. Consequently, FF interferes with the welfare of prostate cancer cells, as well as their expansion and invasiveness [24,25]. Moreover, we have recently shown that FF can also impair the acquired chemoresistance of prostate cancer cells, thus increasing their sensitivity to cytostatic drugs. This effect is apparently exerted through the interference of FF with mitochondrial respiration, which inhibits energy production and impairs the efficiency of drug efflux systems [26]. These data confirm its potential for the palliative treatment of prostate cancer patients; however, cancer cells can adapt to the metabolic block via reprogramming their metabolism [27]. Due to the links between the metabolic profile and invasiveness of cancer cells [9], FF-induced metabolic reprogramming may still have consequences for cancer progression.

Increasing interest in the application of FF and other metabolic blockers in prostate cancer therapy has justified studies on cellular adaptation to the combined chemotherapeutic/metabolic stress [8]. Such studies use an experimental approach based on the prostate cancer cell lineages with acquired chemoresistance [26,28,29]. They have revealed the signs of such adaptation to the combined docetaxel (DCX)/FF treatment, i.e., the activated autophagy, polyploidisation and the function of CD44^+^ stem-like cells [30]. On the other hand, the interrelations between FF sensitivity and the “natural history” of prostate cancer, defined as the sequence of evolutionary events that determine its expansiveness, invasiveness, metabolic profile and drug resistance, remain elusive. They may impair the welfare of hyperlipidemic patients with diagnosed prostate cancer who are natural candidates for long-term FF applications after chemotherapy. Therefore, to fill this gap, we assessed the consequences of the long-term exposition of drug-resistant prostate cancer cells to FF. Accordingly, we analyzed (i) the FF reactivity of originally drug-resistant PC3_DCX20 cells that have undergone “retroevolution” towards a drug-sensitive phenotype. Furthermore, (ii) we exposed wtPC3 and PC_DCX20 cells to a long-term FF treatment in conditions that mimic its differential bioavailability and (iii) comprehensively analyzed their phenotype and FF reactivity.

## 2. Results

### 2.1. Drug Resistance of PC3 Cells Does Not Correlate with Their Sensitivity to Fenofibrate

**Figure 1:**
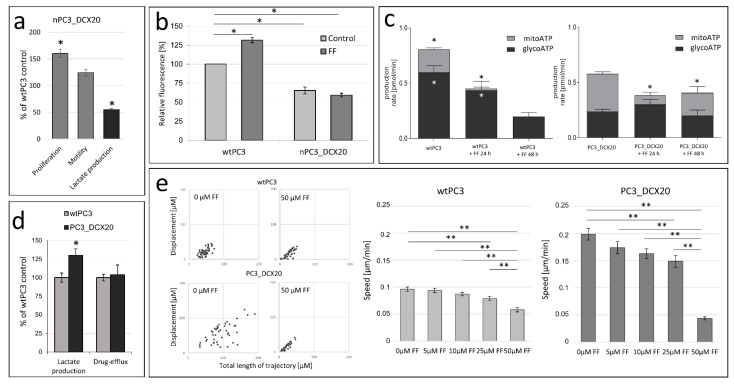
**Loss of drug resistance does not attenuate PC3 sensitivity to fenofibrate.** (**a**) Relative proliferation, motile activity and lactate production in native PC3_DCX20 populations calculated with the Coulter counter, time-lapse videomicroscopy and lactate production assay, respectively, in relation to the wtPC3 control. (**b**) Effect of 25 µM FF on the relative calcein levels in calcein-loaded populations of wtPC3 and native (n)PC3_DCX20 cells (cf. Figure S1a). (**c**) Effect of 25 μM FF on the metabolic profile of wtPC3 (**left**) and nPC3_DCX20 cells (**right**). Analyses of mitochondrial and glycolytic ATP production (mitoATP and glycoATP, respectively) were based on Seahorse analyses of oxygen consumption rate (OCR) and extracellular acidification rate (ECAR), respectively. (**d**) Lactate production and drug efflux intensity in wtPC3 and long-term expanded PC3_DCX20 cells. (**e**) Motility of wtPC3 (**left**) and PC3_DCX20 cells (**right**) in the contol conditions and in presence of FF (5, 10, 25 or 50 µM). Column charts show averaged rate of cell movement calculated at the population level (N > 40). The statistical significance of the differences was estimated with the use of Student’s *t*-test (**a**–**d**) or Kruskal–Wallis test (**e**). * *p* < 0.05; ** *p* ≤ 0.01. Note the retroevolution of nPC3_DCX20 cells towards the low drug efflux phenotype and the low FF tolerance of PC3_DCX20 cells.

Previously, we have shown that the long-term exposition of prostate cancer PC3 cells to docetaxel (DCX) results in the expansion of a DCX-resistant (PC3_DCX20) lineage that displays an increased sensitivity to fenofibrate (FF) [26,30]. A discrete phenotype of “native” DCX-resistant (n)PC3_DCX20 cells was illustrated by their increased proliferation and motility rate, as well as their reduced lactate production in comparison to those of wtPC3 cells (as estimated by cell counting, time-lapse videomiscroscopy and biochemical assays, respectively; Figure 1a). In control conditions, a more efficient calcein efflux could also be seen from nPC3_DCX20 cells than from their wtPC3 counterparts (Figure 1b). This observation demonstrates the relatively high activity of the ABC efflux pumps and the multi-drug resistance of nPC3_DCX20 cells [30]. Seahorse analyses of the oxygene consumption and medium acidification (hallmarks of mitochondrial oxidative phosphorylation (OXPHOS) and glycolysis, respectively) revealed a high OXPHOS intensity in the drug-resistant nPC3_DCX20 cells (Figure 1c). FF efficiently interfered with the drug efflux (Figure 1b) and OXPHOS efficiency in wtPC3 cells (Figure 1c), whereas FF-treated nPC3_DCX20 populations retained a relatively high calcein efflux (Figure 1b) and OXPHOS intensity in the presence of FF (Figure 1c). In conjunction with the relatively high FF sensitivity of nPC3_DCX20 cells [30], these data suggested that the drug efflux systems are not crucial for the FF resistance of prostate cancer cells. Actually, nPC3_DCX20 cells subjected to long-term (5-month) propagation in the absence of any therapeutic stress gave rise to the PC3_DCX20 lineage, which was characterized by an intensified glycolysis and a relatively low drug efflux activity (similar to that estimated for wtPC3 cells; Figure 1d). These cells retained a high FF sensitivity, as illustrated by their impaired motility in the presence of 50 µM (to ca. 25% of control; Figure 1e). Thus, we confirmed the links between drug resistance and the metabolic profile of prostate cancer cells. However, FF sensitivity is not directly linked with the efficiency of conventional drug resistance systems and the cellular metabolic profile.

### 2.2. FF Sensitivity of PC3_DCX20 Cells

**Figure 2:**
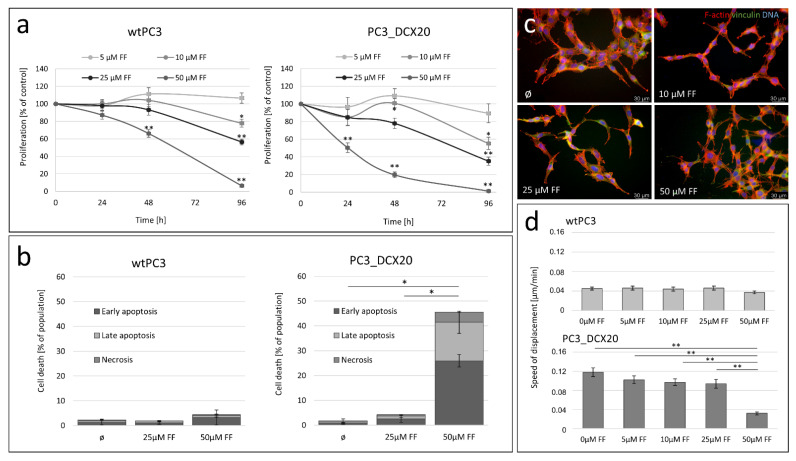
**FF sensitivity of PC3_DCX20 cells.** (**a**) Proliferation of wtPC3 and PC3_DCX20 cells in the presence of FF (5–50 µM) estimated with the Coulter counter at the indicated time-points. (**b**) wtPC3 and PC_DCX20 cells were incubated in the presence of 25 and 50 µM FF for 48 h and their apoptotic response was estimated with the flow cytometry-assisted AnnexinV/Propidium iodide assay (AV^+^/PI^−^: early apoptosis; AV^+^/P^+^: late apoptosis; AV^−^/P^+^: necrosis; N ≥ 5000; cf. Figure S2a). Column plots show percentages of apoptotic and necrotic cells (±SEM). (**c**) Morphology of FF-treated (5–50 μM) PC3_DCX20 cells visualized by actin (red)/vinculin (green)/DNA (blue) staining and immunofluorescence microscopy. (**d**) Displacement of FF (5–50 μM)-treated wtPC3 and PC3_DCX20 cells estimated with time-lapse videomicroscopy (cf. Figure S1b,c). The statistical significance of the differences was estimated with the use of confidence interval of difference of two means (**a**,**b**), or Kruskal–Wallis test (**d**). * *p* < 0.05; ** *p* ≤ 0.01. Note that the attenuated welfare of PC3_DCX20 cells in the presence of 50 μM FF correlates with their high lactate production (cf. Figure 1).

To further scrutinize the consequences of the relatively high FF sensitivity of PC3_DCX20 cells, we compared the proliferation, apoptosis and invasive potential of wtPC3 and PC3_DCX20 cells in the presence of FF. This drug exerted a considerably stronger inhibitory effect on PC3_DCX20 proliferation than on the proliferation of their wtPC3 counterparts (Figure 2a). This effect is especially distinct in the presence of 50 μM FF, in which the wtPC and PC3_DCX20 numbers dropped to 60% and 20% of the control values after 48 h of FF treatment, respectively. Apparently, these differences can be ascribed to the differential pro-apoptotic effect of FF in wtPC3 and PC3_DCX20 populations. A negligible apoptotic response of wtPC3 cells to FF treatment is illustrated by only slightly increased fractions of apoptotic cells in 50 μM FF-treated wtPC3 populations (Figure 2b). Concomitantly, abundant early- and late-apoptotic cells (ca. 27% and 15% of the total, respectively) were seen in PC3_DCX20 populations exposed to 50 µM FF. Their increased incidence was correlated with a lower spread morphology of PC3_DCX20 cells, suggesting their impaired adhesion (especially in the presence of 50 μM; Figure 2c). Along with their impaired translocation (illustrative of lower invasiveness; Figure 2d; cf. Figure S1), these data confirm the therapeutic potential of FF. However, the “natural history of prostate cancer cells” (related to the acquisition of drug resistance and their further retrodifferentiation towards a low-efflux phenotype) can interfere with their FF sensitivity.

### 2.3. Long-Term Fenofibrate Treatment Induces Phenotypic Microevolution of PC3 Cells

**Figure 3:**
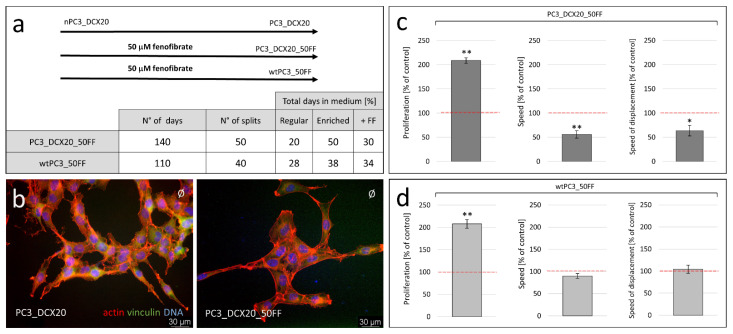
**Fenofibrate-induced phenotypic microevolution of PC3 cells.** (**a**) The scheme of the experimental approach for the establishment of FF-resistant PC3 progenies. (**b**) Morphology of PC3_DCX20 and PC3_DCX20_50FF cells estimated with immunofluorescence (F-actin/vinculin/DNA staining). (**c**) Proliferation and motility parameters of PC3_DCX20_50FF cells incubated in control conditions and estimated with the Coulter counter and time-lapse videomicroscopy, respectively (% of PC3_DCX20 control (100%)). (**d**) Proliferation and motility rates of wtPC3_ 50FF cells (% of wtPC3 control (100%)). The statistical significance of the differences was estimated with the use of confidence interval of quotient of two means (**c**,**d**). * *p* < 0.05; ** *p* ≤ 0.01. Note the phenotypic evolution of PC3 lineages that have undergone long-term FF-induced stress.

Further studies were designed to elucidate whether the long-term FF-induced metabolic imbalance can prompt the reprogramming/microevolution of drug-resistant prostate cancer cells towards an FF-resistant phenotype. For this purpose, we engineered an experimental protocol that mimics permanent prostate tumor exposition to fenofibrate (Figure 3a). A long-term (5 months, ~50 splits), intermittent PC3_DCX20 cultivation in the presence of 50 µM FF prompted the selective expansion of an FF-resistant, phenotypically discrete PC3_DCX20_50FF lineage (for details, see Materials and Methods). When cultivated in the control conditions, the PC3_DCX20_50FF cells displayed a slightly more elongated morphology (Figure 3b), accompanied by a considerably lower motility and ca. a two-fold higher proliferation rate than that of the “maternal” PC3_DCX20 cells (Figure 3c). Corresponding analyses of the wtPC3_50FF lineage, which had been derived from wtPC3 cells, revealed that their proliferation rate again was ca. two-fold higher than that of the naïve cells, in the absence of any distinct differences in motile activity (Figure 3d). Collectively, these data indicate that long-term FF-induced stress can prompt the phenotypic microevolution of prostate cancer cells regardless of their “natural history”.

### 2.4. PC3_DCX20_50FF Cells Display the FF-Resistant Phenotype

**Figure 4:**
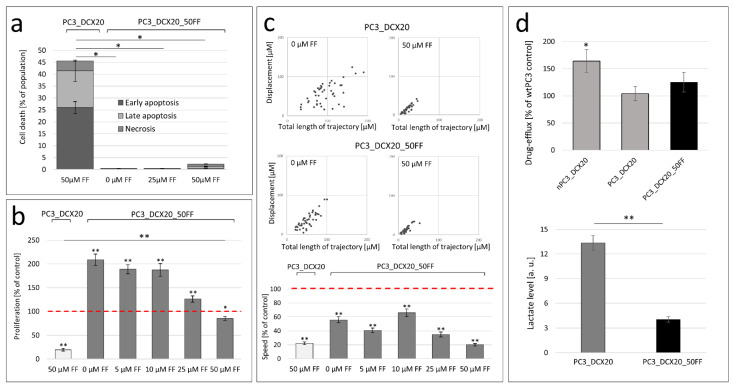
**Sensitivity of PC3_DCX20_50FF cells to fenofibrate.** (**a**) Apoptotic response of PC3_DCX20_50FF cells to 25 and 50 µM FF (48 h) estimated with the flow cytometry-assisted AnV/PI assay (N ≥ 5000, cf. Figure S2b). Column plots show percentages of apoptotic and necrotic cells (±SEM). (**b**) Proliferation of PC3_DCX20_50FF cells in the presence of FF (5, 10, 25 or 50 µM) estimated with the Coulter counter and calculated as % of naïve PC3_DCX20 control (100%). (**c**) Motility of PC3_DCX20_50FF cells in the presence of FF (5, 10, 25 or 50 µM) estimated with time-lapse videomicroscopy 48 h after FF administration and calculated as percentage of naïve PC3_DCX20 control (100%). (**d**) Drug efflux intensity and lactate production in PC3_DCX20_50FF cells estimated with the calcein efflux assay and the lactate secretion assay. The statistical significance of the differences was estimated with the use of confidence interval of difference of two means (**a**), confidence interval of quotient of two means (**b**,**c**), or Student’s *t*-test (**d**). * *p* < 0.05; ** *p* ≤ 0.01. Note the relatively high proliferation rate of PC3_DCX20_50FF cells in the presence of FF.

To estimate the consequences of the FF-induced prostate cancer microevolution, we further evaluated the FF sensitivity of PC3_DCX20_50FF cells. These analyses revealed a very low pro-apoptotic activity of FF in PC3_DCX20_50FF populations. It was illustrated by residual numbers of apoptotic and necrotic PC3_DCX20_50FF cells (considerably less pronounced than in PC3_DCX20 populations; Figure 4a). This difference was especially distinct in the presence of 50 µM FF, in which ca. 5% of the PC3_DCX20_50FF cells were classified as apoptotic/necrotic (compared to ca. 45% in PC3_DCX20 populations). Analyses of PC3_DCX20_50FF proliferation confirmed a weak cytostatic effect of FF (Figure 4b). This is illustrated by a negligible inhibition of PC3_DCX20_50FF proliferation in the presence of 50 µM FF (ca. 55% compared to ca. 80% in PC3_DCX20 populations). The final cytostatic effect of FF was further weakened by the high proliferative activity of PC3_DCX20_50FF cells. Consequently, their proliferation rate in the presence of 50 μM FF corresponded to that of PC3_DCX20 cells in the control conditions and was almost four-fold higher than that estimated for 50 µM FF-treated PC3_DCX20 cells. In turn, a low basic motility of PC3_DCX20_50FF, compared to that of the PC3_DCX20 cells, resulted in their similar motility rates in the presence of 50 μM FF, even though the PC3_DCX20_50FF cells were less sensitive to 50 μM FF (Figure 4c). Concomitantly, the PC3_DCX20_50FF cells displayed a slightly higher drug efflux efficiency and a lower glycolysis rate than the PC3_DCX20 cells (Figure 4d). Corresponding effects were observed in wtPC3_50FF populations (Figure S3). Thus, we show that the long-term exposition to high FF concentrations facilitates a selective expansion of FF-resistant PC3 lineage(s). These observations further prompted us (i) to address the effects of FF bioavailability on the PC3 microevolution pattern and (ii) to elucidate the consequences of FF-induced microevolution for the formation of an invasive front.

### 2.5. Fenofibrate Bioavailability Affects PC3 Microevolution Pattern

**Figure 5:**
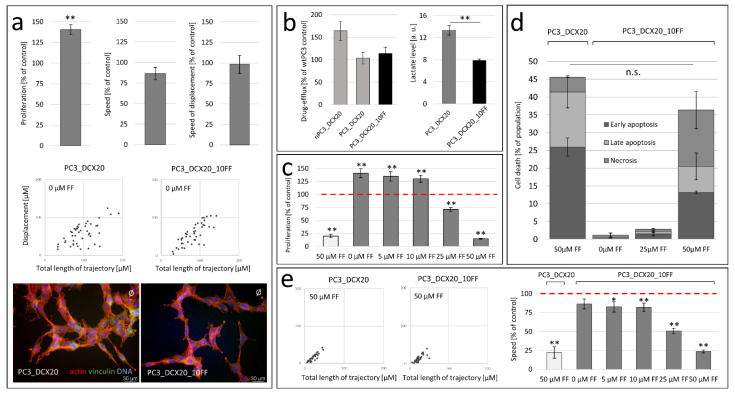
**Phenotypic properties of PC3_DCX20_10FF cells.** (**a**) Proliferation, motility rates and cytoskeleton architecture PC3_DCX20_10FF cells incubated in control conditions and estimated with the Coulter counter, time-lapse videomicroscopy and immunofluorescence (F-actin/vinculin staining) in relation to PC3_DCX20 cells. (**b**) Drug efflux intensity and glycolysis intensity of PC3_DCX20_10FF cells (close bars) estimated with the calcein efflux assay and the lactate secretion assay. (**c**,**d**) Proliferation (**c**) and apoptotic responses ((**d**), cf. Figure S2c) of PC3_DCX20_10FF cells to FF estimated with Coulter counter (% of PC3_DCX20 control (100%) and flow cytometry, respectively. (**e**) Motility of PC3_DCX20_10FF cells in the presence of FF (5, 10, 25 or 50 µM) calculated as % of PC3_DCX20 control (100%). The statistical significance of the differences was estimated with the use of confidence interval of quotient of two means (**a**,**c**,**e**) or confidence interval of difference of two means (**b**,**d**). * *p* < 0.05; ** *p* ≤ 0.01, n.s.: no statistical difference. Note the FF sensitivity of PC3_DCX20_10FF cells.

To simulate prostate cancer microevolution under a less intense FF stress, we subjected PC3_DCX20 cells to a long-term 10 µM FF treatment (cf. Figure S4). Consequently, a phenotypically discrete PC3_DCX20_10FF lineage was established, which displayed an enhanced proliferation, similar translocation (Figure 5a) and reduced lactate secretion in comparison to those of PC3_DCX20 cells (Figure 5b). This phenotypic drift was accompanied by a modified reactivity of PC3_DCX20_10FF cells to FF, illustrated by the induction of their proliferation in the presence of 10 μM FF and by their relatively low tolerability to higher FF concentrations (Figure 5c). The pro-apoptotic activity of 50 µM FF was considerably higher in PC3_DCX20_10FF than in PC3_DCX20_50FF populations (Figure 5d), although it was lower than that in PC3_DCX20 populations. Corresponding effects of FF on PC3_DCX20_10FF motility (Figure 5e) indicate that 10 μM FF is still able to induce the adaptative responses of PC3 cells. This notion is supported by the consequences of the long-term 10 μM FF treatment for the phenotype of wtPC3 cells (in particular, the relatively high motility of wtPC3_10FF cells in the absence/presence of FF; Figure S5). However, a reduced FF bioavailability may affect the direction of cell microevolution, potentially accounting for the phenotypic diversification of cell lineages within prostate tumors.

### 2.6. Long-Term FF Treatment Does Not Prompt the Invasiveness of PC3 Cells

**Figure 6:**
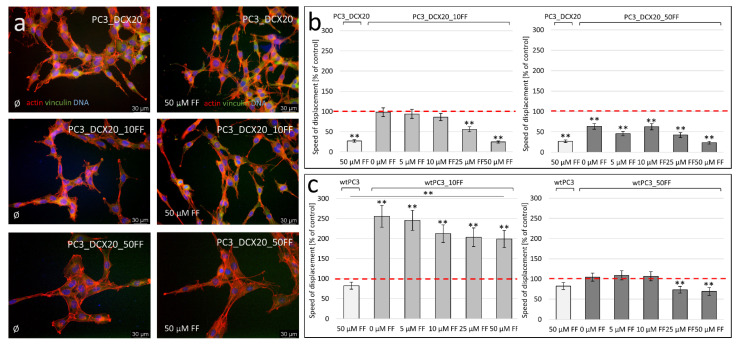
**Long-term fenofibrate effects on the invasiveness of PC3 cells.** (**a**) Morphology of PC3_DCX20, PC3_DCX20_10FF and PC3_DCX20_50FF cells, estimated with immunofluorescence microscopy (vinculin: green; F-actin: red; Scale bar = 30 μm). (**b**,**c**) Effect of FF on the invasiveness (displacement rates) of PC3_DCX20 (**b**) and wtPC3 lineages (**c**) estimated with time-lapse videomicroscopy (% of arelevant control (100%)). The statistical significance of the differences was estimated with the use of confidence interval of quotient of two means (**b**,**c**). ** *p* ≤ 0.01. Note the diverse short- and long-term effects of FF on cell invasiveness.

To estimate the effect of FF-induced microevolution and FF bioavailability on the formation of the tumor invasive front, we looked into the invasive potential of PC3 lineages that have undergone FF-induced microevolution. For this purpose, we traced the morphology and displacement rates of PC3_DCX20_10FF and PC3_DCX20_50FF cells under short-term FF stress. No significant differences in the morphology, rear-front polarisation and overall cytoskeleton architecture could be seen between these lineages in the control conditions and in the presence of 50 μM FF (Figure 6a). Furthermore, PC3_DCX20_10FF and PC3_DCX20_50FF cells reacted to the short-term FF treatment with a considerable attenuation of their displacement rates (Figure 6b). These data suggest the negligible effects of FF-induced microevolution on the invasiveness of prostate cancer cells. Corresponding analyses of wtPC3 lineages revealed the relatively high invasiveness of wtPC3_DCX10 cells both in the absence and in the presence of FF (Figure 6c). Thus, an attenuated FF bioavailability can have profound consequences for the progression of the metastatic cascade and for the recruitment of discrete cell lineages to the invasive front of prostate cancer.

## 3. Discussion

Early speculations on the application of FF in anti-cancer therapy were prompted by its antioxidant, anti-inflammatory and antifibrotic activities. The cytostatic effects of FF were seen in in vitro melanoma, neuroblastoma, myeloma/lymphoma and fibrosarcoma models as well as in breast, oral, liver, glioma, prostate, pancreas and lung cancer cell lines [10,21,31]. They are manifested by the inhibition of cell proliferation and by the invasiveness that has been detected in the presence of FF applied at physiologically relevant concentrations (25–50 μM). Additionally, FF impairs cancer cell viability and induces the pro-apoptotic responses of cancer cells [10,20,24,32,33,34]. These effects can be exerted via its “canonical” effector (PPARα) or through the impairment of mitochondrial homeostasis. Recent findings on FF’s interference with the drug resistance of cancer cells [26] have prompted speculations on the possible introduction of FF into the palliative treatment of advanced prostate tumors. However, the combined application of docetaxel and FF has also been shown to invoke adverse effects. In the case of prostate cancer, they are related to the selective survival of CD44-positive stem-like cells and to the further propagation of their DCX-(hyper)resistant progenies [30]. Here, we continued this line of research to estimate the consequences of acquired drug resistance for the FF-induced phenotypic microevolution of prostate cancer cells. Our data demonstrate that the “natural history” of prostate cancer cells affects their sensitivity to FF in a manner independent to the “conventional” drug efflux systems and to their metabolic profile. On the other hand, (ii) FF directs the pattern of phenotypic microevolution towards expansive, FF-resistant phenotype(s) and (iii) this effect is dependent on FF bioavailability. Thus, we identify potential adverse effects of FF-based metabolic therapies that follow the traditional prostate cancer chemotherapy.

Single-cell adaptation responses to the microenvironmental (cell niche-dependent) signalling and the clonal expansion of their progenies result in the phenotypic microevolution of cancer. The selective pressure exerted by cytostatic drugs has long been known to cause similar effects [35,36]. They account for the low efficiency of prostate cancer treatment strategies and malignant tumor relapses after chemotherapy [37,38,39]. Surprisingly, the enhanced drug efflux efficiency of chemo-resistant prostate cancer cells, which underlies this effect, has previously been shown to correlate with their FF sensitivity. DU145 lineages, which have been derived from wtDU145 cells upon their long-term exposition to increasing DCX concentrations (DU145_DCX20 and DU145_DCX50 cells), displayed an enhanced sensitivity to FF [26,30]. The relatively high FF sensitivity of drug-resistant nPC3_DCX20 cells confirms that the activity of ABC transporters is neither the prerequisite nor the determinant of FF resistance. This notion was further supported by a relatively high FF sensitivity of PC3_DCX20 cells after their retroevolution towards a drug efflux^low^ phenotype. On the other hand, both the DU145_DCX20 (unpublished data) and nPC3_DCX20 cells displayed the OXPHOS^high^/glycolysis^low^ metabolic profile. This might indicate the interrelation between the OXPHOS intensity and FF sensitivity of prostate cancer cells. FF activity is commonly related to PPARα activation [40,41,42]. However, FF can also interfere with the activity of fatty acid synthase, impair mitochondrial respiration and induce mitochondrial ROS accumulation (in a PPARα-independent manner) [43]. FF-induced β-oxidation, the impairment of intracellular ATP production and the activation of AMPK/the inhibition of mTOR activity have been reported [20]. The interference of FF with the OXPHOS could therefore induce mitochondrial dysfunction and oxidative stress in OXPHOS-addicted cells [20,25,44,45]. On the other hand, the retroevolution of PC3_DCX20 cells towards a drug-efflux^low^ phenotype also enhanced their glycolysis but did not erase a high FF sensitivity. Apparently, the susceptibility of mitochondria to FF-induced stress rather than OXPHOS activity determines the sensitivity of drug-resistant prostate cancer cells to the short-term FF treatment [46].

Studies performed to trace the long-term consequences of FF-induced stress have demonstrated that the FF sensitivity of PC3_DCX20 cells did not disturb their ability to adapt to the long-term FF-treatment. Actually, we are the first to show that FF can prompt the phenotypic microevolution of prostate cancer cells towards the FF-resistant phenotype. This is illustrated by the relatively high proliferation rate of PC3_DCX20_50FF cells in the control conditions, accompanied by their sustained proliferation and negligible apoptosis in the presence of 50 μM FF. Additionally, the FF-induced inhibition of motility/invasiveness was less pronounced in PC3_DCX20_50FF populations. However, our data indicate that the long-term FF application at physiologic concentrations (<100 μM) is sufficient to enhance their expansive potential. This notion was further confirmed by the corresponding properties of wtPC3_50FF cells, which had been derived from the long-term FF-treated wtPC3 cells. Finally, the glycolysis^low^ phenotype of FF-resistant PC3_DCX20_50FF cells, in conjunction with the glycolysis^high^ phenotype of FF-sensitive PC3_DCX20 cells, indicates that the aerobic respiration intensity does not directly determine their FF sensitivity. The drug-efflux^low^ phenotype of PC3_DCX20_50FF cells also confirms that the activity of drug efflux systems is neither necessary nor sufficient for their FF resistance. These observations remain in concordance with the reports on the effective adaptation of (prostate) cancer cells to long-term metabolic stress [26,30]. Apparently, the long-term fenofibrate treatment prompts the microevolution of prostate cancer cells towards an expansive phenotype in a manner dependent on the mitochondrial stress adaptation.

When extrapolated to an in vivo situation, the FF-induced acquisition of a resistant and expansive phenotype by prostate cancer cells may have profound consequences for prostate cancer promotion. First, it facilitates tumor growth, whereas the attenuated motility and proliferation of FF-treated wtPC3_50FF and PC_DCX20_50FF cells can represent an adaptative response to the FF-induced stress. An open question remains of how exactly tumor hyperplasia affects the intratumoral FF bioavailability; however, it is conceivable that FF concentrations within a fast-growing tumor may be significantly reduced. Consequently, the pattern of phenotypic microevolution of prostate cancer cells is modified, as illustrated by rather significant differences between PC3_DCX20 lineages established after the long-term 50 μM and 10 μM FF treatments. PC3_DCX20_10FF cells display a relatively lower proliferative potential, but higher lactate production and motile activity than PC3_DCX20_50FF cells. Concomitantly, they were more susceptible to the cytostatic FF activity than their PC3_DCX20_50FF counterparts. A similar dose-dependence of the FF-induced microevolution pattern was observed in wtPC3-derived lineages, in which we also noticed the considerably increased motility/invasiveness of wtPC3_10FF cells both in the absence and presence of FF. Conceivably, the local bioavailability of FF can sculpt the niche-specific microevolution pattern(s) of individual cell lineages that further determine their recruitment to the invasive front(s) of tumors. Thus, subtle differences in the activity of metabolic blockers may account for the highly diversified biological and clinical scenarios.

Collectively, we demonstrate that the long-term exposition of prostate cancer cells to FF can prompt their phenotypic microevolution towards an expansive, FF-resistant phenotype. At the moment, it is premature to judge whether phenotypic adaptation (epigenetic adaptative expansion) or Darwinian “survival of the fittest cell” (i.e., selective expansion) accounts for the FF-induced phenotypic microevolution of prostate cancer cells. The precise mechanisms of the effect of long-term FF treatment on mitochondrial adaptation processes and cell line specificity of FF-induced phenotypic evolution also require further study. However, it is unlikely that the pattern of this microevolution will always be the same. Since we observed poly(morpho)nuclear giant cells in FF-treated PC3 populations, it may well be that they can be involved in this process [47]. Nevertheless, our data indicate that FF can stimulate and/or redirect the microevolution of prostate tumors. They are added to the list of the potential adverse effects of FF, which also include an increase in oxidative stress, the mediation of mtDNA damage and potential liver tumor promotion [48,49]. They should be carefully considered as crucial for the safety of the palliative treatment of (prostate) cancer patients. At the methodological level, we also show the potential of an experimental approach based on the long-term FF-stress adaptation for the in vitro analyses of the adverse effects of common drugs. Nowadays, prostate cancer patients are routinely subjected to combined or consecutive anti-cancer and anti-hyperlipidemic regimes. Our findings signal the need for a reconsideration of FF application (and the application of other metabolic blockers) in their palliative treatment.

## 4. Materials and Methods

### 4.1. Cell Cultures

Human prostate carcinoma PC3 cells (ATCC^®^ CRL-1435™1) were cultivated in DMEM/F12 HAM medium (Sigma, St. Louis, MO, USA) with supplements (10% FBS) and antibiotics (a regular medium). For the establishment of DCX-resistant PC3_DCX20 lineage, PC3 cells were subjected to the consecutive DCX treatment/recovery cycles, based on their intermittent exposition to DCX administered at increasing concentrations (1, 2, 5, 10, 20 nM) of DCX [26,30]. FF-resistant PC3/PC3_DCX20 sub-lineages were established using a protocol based on the consecutive 10/50 μM FF treatment/recovery cycle (2–3 day-long exposition to FF followed by cell recovery in the fresh regular medium or the enriched medium—DMEM/F12 HAM medium supplemented with 20% FBS (and antibiotics); cf. Figure 3 and Figure S4). For endpoint experiments, media supplemented with FF were added to cancer cell cultures at the concentrations and time points indicated in the text (5–50 μM; F6020, Sigma). These concentrations correspond to its serum concentrations in vivo.

### 4.2. Proliferation and Apoptosis Assay

Cells were seeded into 24-well plates (Corning, Corning, NY, USA) at the density of 5 × 10^3^ cells/cm^2^ and were cultivated for 24 h before the application of control/FF-containing media. After 48 h, the cells were harvested, re-suspended in the original culture medium and counted with the Coulter Z2 Counter (Beckman Coulter Inc., Fullerton, CA, USA). For estimation of apoptosis, the cells were harvested, re-suspended in the original medium and subjected to the AnnexinV/propidium iodide staining according to the manufacturer’s protocol (BD Pharmigen, San Diego, CA, USA). Flow cytometric detection of apoptotic cells was performed with the FACSAria FACS system (Becton–Dickinson, Heidelberg, Germany [26]). At least 5 × 10^3^ cells were analyzed for each condition.

### 4.3. Cell Motility

Cells were seeded at the density of 1000 cells/cm^2^ and incubated for 24 h. Then, the cells were subjected to FF, cultivated for 48 h and time-lapse analyses were performed. Cell movement was estimated with the time-lapse Leica DMI6000B videomicroscopy system equipped with a temperature chamber (37 ± 0.2 °C)/(5% CO_2_), the IMC contrast optics and a cooled, digital DFC360FX CCD camera. Cell trajectories were constructed from the sequences of centroid positions recorded for 8 h at 300 s time intervals (×10, NA 0.75 objective) to calculate single-cell movement parameters: the speed of cell movement (motility; i.e., total length of single-cell trajectory/time of registration (μm/min) and the speed of cell displacement (displacement; μm/min). Data from 45 trajectories (≥3 independent experiments) were then pooled and analyzed to calculate cell movement parameters at the population level [3,50].

### 4.4. Lactate Production Assay

Cells were seeded into 96-well plates (Eppendorf, Hamburg, Germany) at the density of 5 × 10^3^ cells/well, incubated for 24 h and treated with FF for 48 h. Intracellular lactate contents were estimated using the lactate assay kit (cat. no. MAK064; Sigma-Aldrich, St. Louis, MO, USA) according to the manufacturer’s protocol. Cells were lysed and the cell lysates were transferred to white plates for the collection of luminescence signals with the Infinite M200 reader. Lactate contents per 10^5^ cells were calculated to estimate lactate production at the single-cell level. 570 nm absorbance was measured with the MultiskanTM FC Microplate Reader (ThermoFisher Scientific, Waltham, MA, USA).

### 4.5. Calcein Efflux Assay

For the estimation of drug efflux efficiency, calcein efflux assay was performed. CalceinAM (Invitrogen, Waltham, MA, USA, No. C3099) was administered at the concentration of 1 µg/mL in FluoroBrite^®^ DMEM (supplemented with 10% FBS and 1% GlutaMAX). Monitoring of free calcein fluorescence intensity was performed with the Leica DMI6000B fluorescence microscope (Alexa488 filter set and time-lapse imaging module (time step = 5 min)). The changes of this parameter over time were used to illustrate the efficiency of drug efflux [17,21]. For the fluorimetric analyses of calcein-stained specimens, the stacks of fluorescence images of at least 16 randomly chosen confluent culture regions were collected. They were registered with the same excitation/exposure settings (excitation/camera gain/time of exposition). Where indicated, the averaged difference between calcein fluorescence at the beginning and end of experiment (drug efflux intensity) was calculated as the percentage of relevant control.

### 4.6. Seahorse

Cells were plated at the optimal densities in the Seahorse XF 8-well plates 48/72 h before the measurement, subjected to the FF treatment for 24/48 h and incubated in the Seahorse XF Assay Media at 37 °C for 1 h without CO_2_, immediately before starting the Real-Time ATP Rate Assay (Seahorse Bioscience; North Billerica, MA, USA, 1 μM Oligo; 1 μM/0.5 μM Rot/AA). OCR and ECAR measurements were performed with the Seahorse Analyzer XF HS Mini/XFp software and normalized to cell numbers.

### 4.7. Immunofluorescence

Actin cytoskeleton was visualized in formaldehyde/Triton X-100 fixed/permeabilized cells (FA; 3.7%; 20 min in RT)/Triton X-100 (0.1%; 10 min in RT). After the incubation in the presence of 3% BSA, primary mouse anti-vinculin IgG (No. V9131, Sigma) was applied for 1 h. Afterwards, the cells were labelled with Alexa 488-conjugated goat anti-mouse IgG (No. A11001, Invitrogen, Carlsbad, CA, USA), and counterstained with TRITC-conjugated phalloidin (No. 49409 and 77418, Sigma) and Hoechst 33258 (No. B2883, Sigma). Image acquisition was performed with the Leica DMI6000B microscope (DMI7000 version; Leica Microsystems, Wetzlar, Germany) equipped with the Total Internal Reflection Fluorescence (TIRF) and the Nomarski Interference Contrast (DIC) modules. A 40× NA1.47 oil immersion objective and 14-bit Hamamatsu 9100-02 EM-CCD camera were controlled by LAS-AF 3.4 operation and deconvolution software [24].

### 4.8. Statistical Analysis

The statistical analysis of the data was performed in the Statistica software (13.3 version, 1984–2017 TIBCO Software Inc.; accessed through May 2021–September 2022) With the Student’s *t*-test or Kruskal–Wallis test. Confidence interval of quotient of two means or confidence interval of difference of two means were computed with the use of the GraphPad QuickCalcs calculator [https://www.graphpad.com/quickcalcs/errorProp1/; accessed through May 2021–September 2022]; * *p* < 0.05; ** *p* ≤ 0.01. All data are representative for at least 3 independent experiments. Error bars illustrate ±SEM (standard error of the mean) or ±SD (standard deviation) values (as indicated in the legends).

## 5. Conclusions

We present the data which confirm the activity of fenofibrate as a selective factor that can stimulate and/or redirect the microevolution of prostate cancer. The “conventional” drug resistance systems in prostate cancer cells are not effective against this activity; instead, FF can prompt the phenotypic microevolution of prostate cancer towards an expansive, FF-resistant phenotype in a manner dependent on the intratumoral FF bioavailability. Our data collectively indicate that the subtle effects of metabolic blockers on the phenotype of prostate cancer cells may result in highly diversified biological and therapeutic consequences. They account for the phenotypic diversification of the cells within prostate tumors and can potentially lead to the differential contribution of individual cell lineages to the formation of an invasive front. This notion prompts a need to reconsider the application of fenofibrate and other metabolic blockers in the palliative treatment of prostate cancer patients.

## Data Availability

Data is contained within the article and Supplementary Material.

## Supplementary Materials

The following supporting information can be downloaded at: https://www.mdpi.com/article/10.3390/ph15111320/s1, Figure S1: Sensitivity of wtPC3 and drug-resistant PC3_DCX20 cells to fenofibrate; Figure S2: Pro-apoptotic activity of FF; Figure S3: Sensitivity of wtPC3_50FF cells to fenofibrate; Figure S4: Phenotypic microevolution of PC3_DCX20 and wtPC3 cells under 10 μM FF-induced stress; Figure S5: Phenotypic properties of wtPC3_10FF cells.
