# Supplementary material for: Long-Term Fenofibrate Treatment Stimulates the Phenotypic Microevolution of Prostate Cancer Cells In Vitro
Source: Pharmaceuticals (Basel). 2022 Oct 26;15(11):1320. doi: 10.3390/ph15111320 (PMC9694160; doi:10.3390/ph15111320)
Supplement: Supplementary file 1 [file pharmaceuticals-15-01320-s001.zip › pharmaceuticals-1951470-supplementary.pdf]

# Long-term fenofibrate treatment stimulates the phenotypic microevolution of prostate cancer cells *in vitro*

**Karolina W. Warzecha[1], Maciej Pudełek[1], Jessica Catapano[1], Zbigniew Madeja[1], Jarosław Czyż[1,*]**

**Department of Cell Biology, Faculty of Biochemistry, Biophysics and Biotechnology, Jagiellonian University, ul. Gronostajowa 7, 30-387 Kraków, Poland**

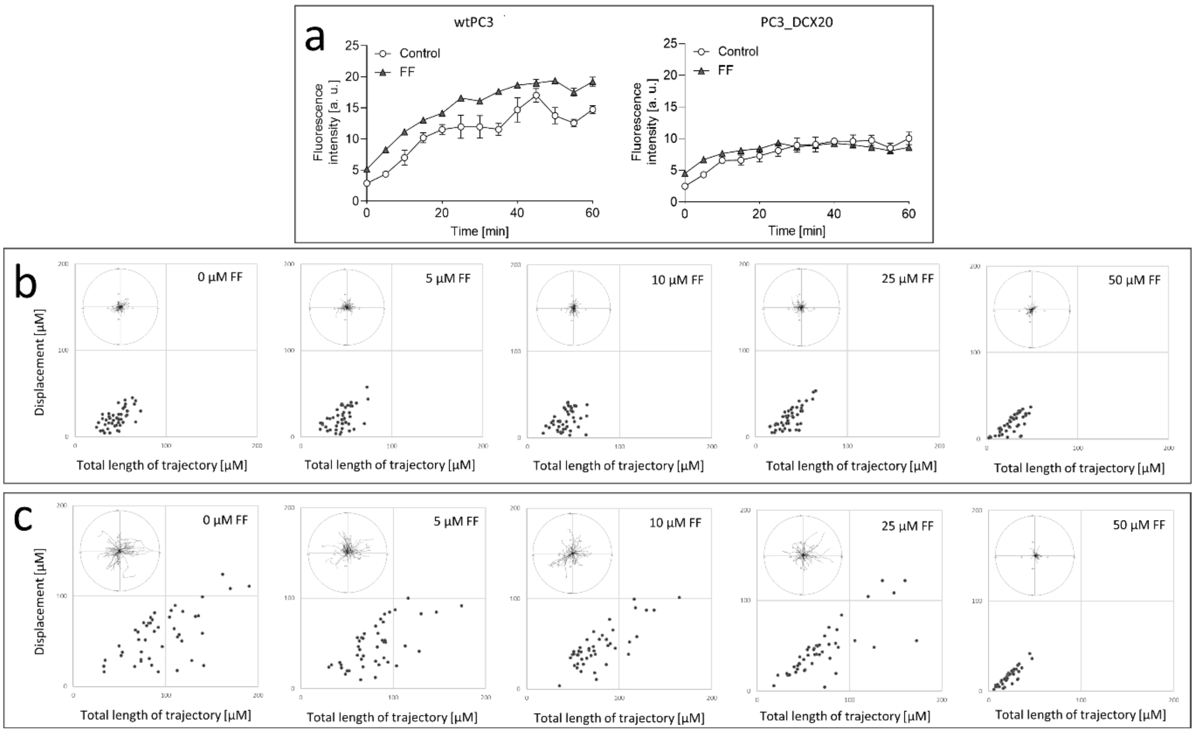


**Figure S1. Sensitivity of wtPC3 and drug-resistant PC3_DCX20 cells to fenofibrate.** (**a**) Representative kinetics of calcein-specific fluorescence intensity following the loading of wtPC3 and PC3_DCX20 cells with calceinAM. **(b, c)** Motility of wtPC3 **(b)** and drug-resistant PC3_DCX20 cells **(c)** incubated in the presence of FF (5, 10, 25 or 50 μM) for 48 hours estimated with time-lapse videomicroscopy (cf. Fig. 1). Cell trajectories are depicted as circular diagrams (axis scale in μm) drawn with the initial point of each trajectory placed at the origin of the plot (registered for 8 hours; N = 45). Dot-plots show movement parameters (total length of cell trajectory and displacement) of single wtPC3 and PC3_DCX20 cells. **Note an increased motility and relatively high sensitivity of PC3_DCX20 cells to FF.**

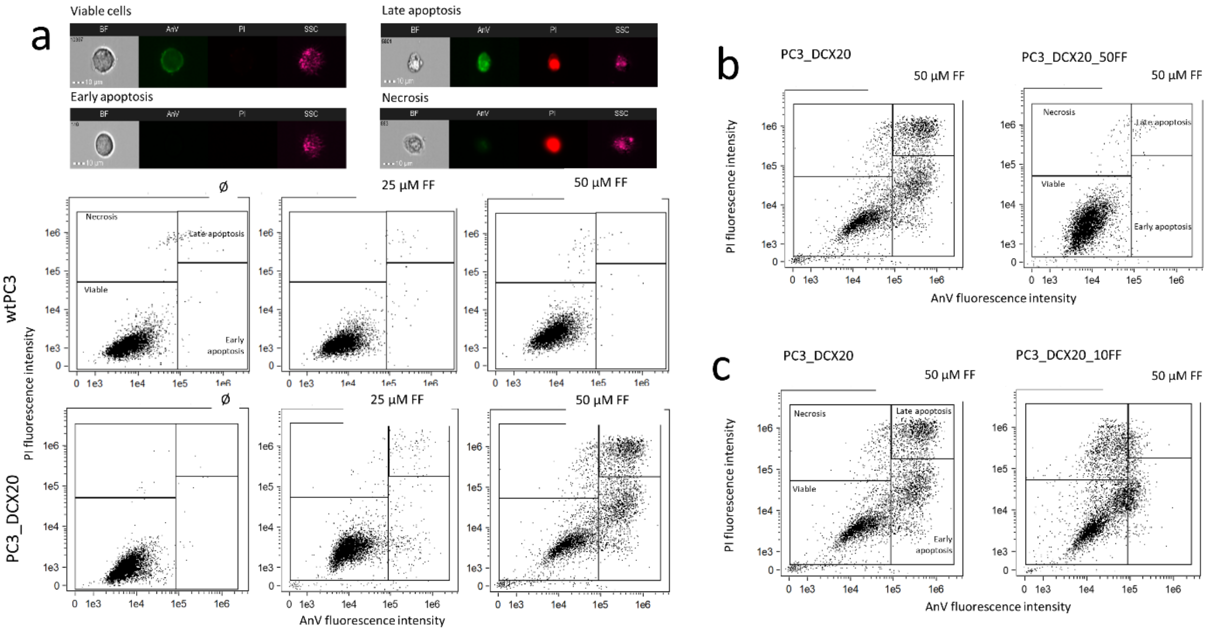


**Figure S2. Pro-apoptotic activity of FF.** wtPC3, PC3_DCX20 **(a)**, PC3_DCX20_50FF **(b)** and PC3_DCX20_10FF cells **(c)** were cultivated in the presence of 25 and/or 50 μM FF and the numbers of apoptotic (AnV/PI-positive) cells were estimated with the flow cytometry-assisted AnV/PI assay (N≥5000). **Note a relatively weak apoptotic response of PC3_DCX20_50FF cells to 50 μM FF.**

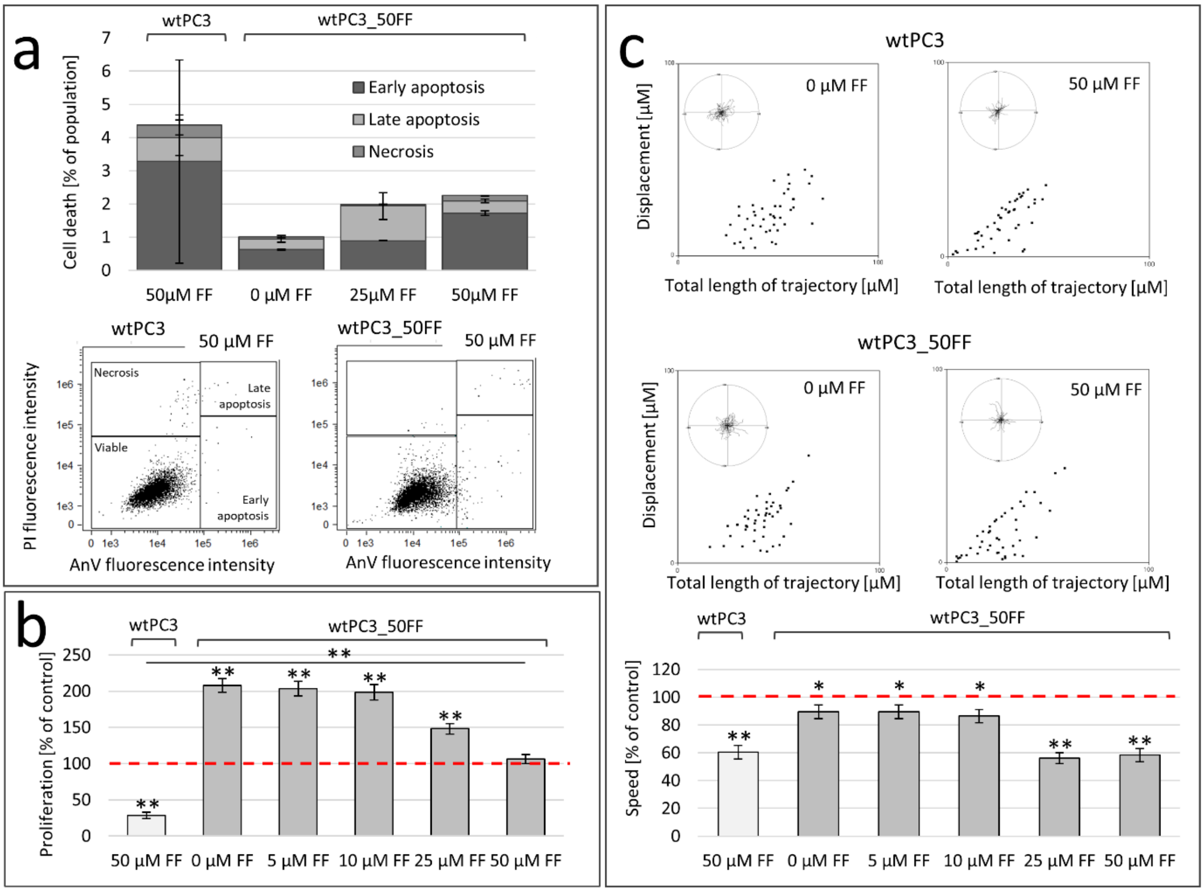


**Figure S3. Sensitivity of wtPC3_50FF cells to fenofibrate.** (**a**) Apoptotic responses of wtPC3_50FF cells to 10 and 50 µM FF (48 hours) estimated with the flow cytometry-assisted AnV/PI assay (N≥5000). Column plots show % of apoptotic and necrotic cells (± SEM). **(b)** Proliferation of wtPC3_50FF cells in the presence of FF (5, 10, 25 or 50 µM) estimated with the Coulter counter and calculated as % of wtPC3 control. **(c)** Motility of wtPC3_50FF cells in the presence of FF (5, 10, 25 or 50 µM) estimated with time-lapse videomicroscopy 48 hours after FF administration and calculated as % of naïve wtPC3 control. The statistical significance of the differences was estimated with the confidence interval of difference of two means (a) and confidence interval of quotient of two means (b, c). *p<0.05; **p≤0.01. **Note a relatively high proliferation rate of wtPC3_50FF cells in the presence of FF.**

**a**

| | | | Total days in medium [%] | | |
|---|---|---|---|---|---|
| | N° of days | N° of splits | Regular | Enriched | + FF |
| PC3_DCX20_10FF | 140 | 50 | 5 | 1 | 94 |
| wtPC3_10FF | 140 | 50 | 5 | 1 | 94 |

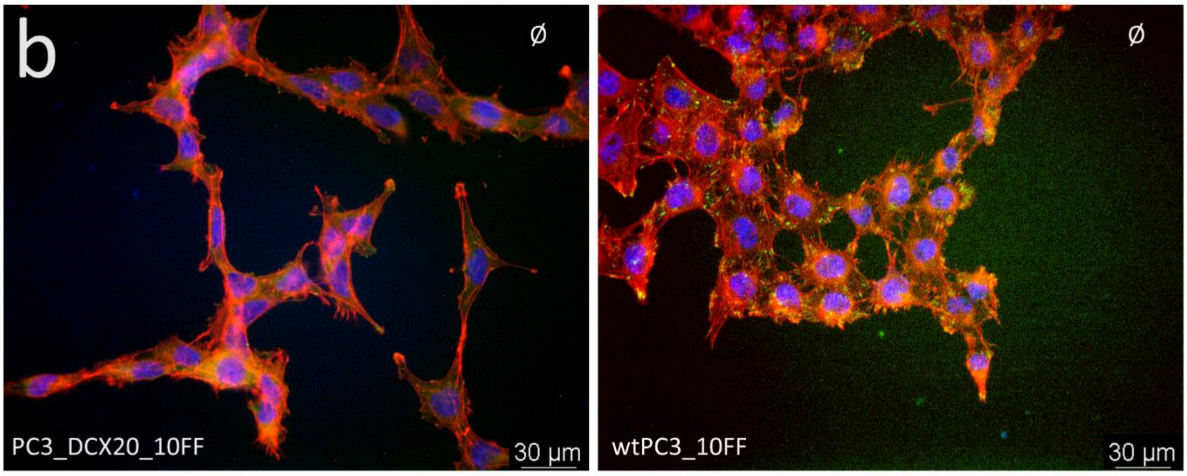


**Figure S4. Phenotypic microevolution of PC3_DCX20 and wtPC3 cells under 10 µM FF-induced stress.** (**a**) Schematic representation of the experimental approach for the establishment of 10 µM FF-resistant cell progenies. (**b**) Morphology of PC3_DCX20_10FF and wtPC3_10FF cells estimated with immunofluorescence microscopy (F-actin/vinculin/DNA staining).

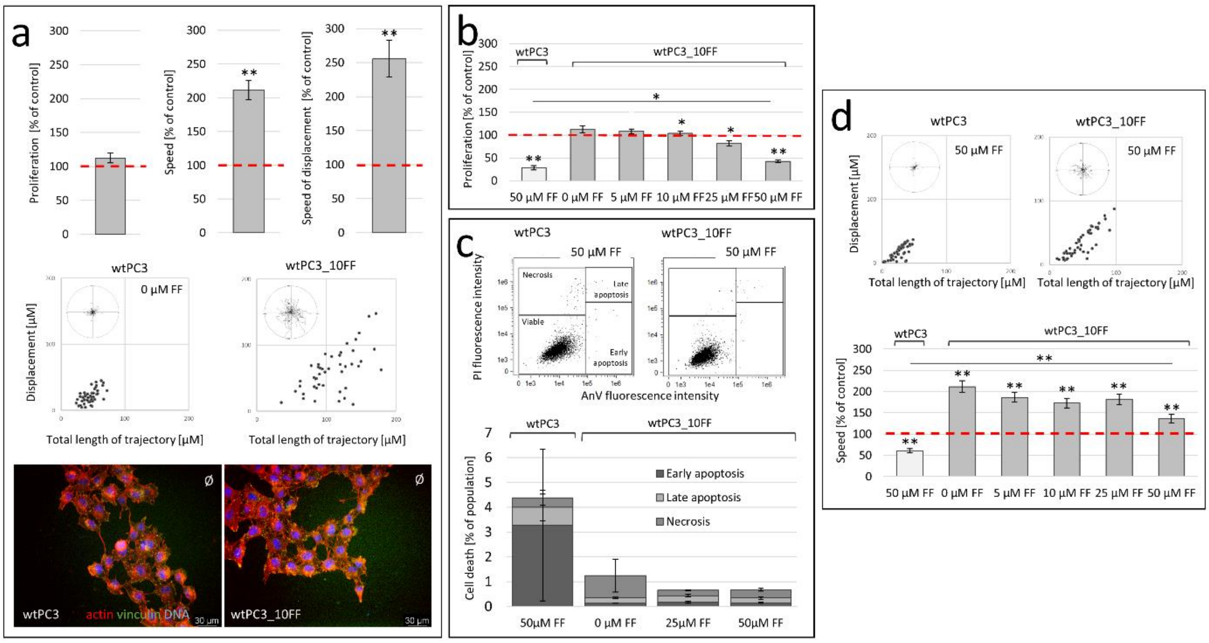


**Figure S5. Phenotypic properties of wtPC3_10FF cells.** (**a**) Proliferation, motility rates and cytoskeleton architecture of wtPC3_10FF cells incubated in control conditions and estimated with the Coulter counter, time-lapse videomicroscopy and immunofluorescence (F-actin/vinculin staining). **(b, c)** Proliferation **(b)** and apoptotic responses **(c)** of wtPC3_10FF cells to FF estimated with Coulter counter and flow cytometry, respectively. **(d)** Motility of wtPC3_10FF cells in the presence of FF (5, 10, 25 or 50 µM) calculated as the % of wtPC3 control. The statistical significance of the differences was estimated with the use of confidence interval of quotient of two means (a, c) or confidence interval of difference of two means (b, d). *p<0.05; **p≤0.01. **Note a relatively high motility of wtPC3_10FF cells in the presence of FF.**
